# Placental Abruption and Partial Placental Prolapse During Labor Induction With a Cervical Double Balloon (Cook Catheter) in a 37-Year-Old Patient

**DOI:** 10.7759/cureus.104391

**Published:** 2026-02-27

**Authors:** Vittoria Olivieri, Piera Caro, Maria Rosaria Pagano, Claudia Casella, Attilio Di Spiezio Sardo

**Affiliations:** 1 Department of Obstetrics and Gynaecology, Azienda Ospedaliera Universitaria Federico II, Naples, ITA; 2 Department of Public Health, School of Medicine, University of Naples Federico II, Naples, ITA; 3 Department of Advanced Biomedical Sciences, School of Medicine, University of Naples Federico II, Naples, ITA

**Keywords:** cervical double balloon, labor induction, placental, placental abruption, placental prolapse

## Abstract

Placental abruption is a major obstetric emergency. To date, no cases of placental abruption associated with partial placental prolapse during labor induction with a cervical double-balloon catheter have been reported. We describe the case of a 37-year-old nulliparous woman (G2P0010) with a pregnancy conceived by assisted reproductive technology and complicated by late-onset fetal growth restriction. At 39 weeks of gestation, labor induction was initiated using a cervical double-balloon catheter because of an unfavorable cervical status. Shortly after insertion, the patient developed acute placental abruption with partial prolapse of a posteriorly inserted placenta, requiring emergency cesarean delivery. This case highlights that placental abruption may occur in temporal association with mechanical cervical ripening and underscores the potential for delayed diagnosis when a cervical double-balloon catheter is in place, as early clinical signs such as vaginal bleeding may be partially obscured.

## Introduction

Placental abruption complicates approximately 0.5% of pregnancies, and it is associated with significant maternal and fetal morbidity and mortality, particularly when diagnosis is delayed [1]. Clinical presentation is often heterogeneous and may include abdominal pain, vaginal bleeding, uterine hypertonus, and signs of maternal hemodynamic compromise. Although placental abruption may occur in otherwise uncomplicated pregnancies [2], it is more frequently associated with hypertensive disorders or abdominal trauma [3].

Mechanical cervical ripening is commonly used for labor induction in the presence of an unfavorable cervix. Cervical double-balloon catheters promote cervical dilation through the application of pressure by an intrauterine and a vaginal balloon, facilitating mechanical dilation, separation of the fetal membranes, and endogenous prostaglandin release, while maintaining stable positioning within the cervical canal [4].

We report a case of acute placental abruption occurring during mechanical induction of labor with a cervical double-balloon catheter in a nulliparous patient with late-onset fetal growth restriction. This case aims to highlight the potential for delayed recognition of placental abruption when mechanical induction methods are used, as the presence of a cervical double balloon may partially obscure early clinical signs, particularly vaginal bleeding.

## Case presentation

A 37-year-old Caucasian nulliparous woman with a history of one first-trimester miscarriage and long-standing infertility presented at 38+6 weeks of gestation with mild, irregular uterine contractions. The pregnancy had been conceived by intracytoplasmic sperm injection and was uncomplicated except for late-onset fetal growth restriction. First-trimester screening showed normal nuchal translucency (1.5 mm) and a low-risk non-invasive prenatal testing result. Second-trimester ultrasound demonstrated fetal biometry appropriate for gestational age and a posteriorly inserted placenta.

On admission, maternal vital signs were within normal limits, and the patient denied vaginal bleeding or leakage of fluid. Vaginal examination revealed a closed, posterior cervix. Ultrasound confirmed a cephalic presentation, an estimated fetal weight below the first percentile, normal amniotic fluid volume, normal umbilical artery Doppler velocimetry with preserved end-diastolic flow (pulsatility index 1.1), and a posteriorly inserted placenta. Given the presence of late-onset fetal growth restriction at term, hospital admission for induction of labor was recommended.

The following day, after cardiotocography confirmed fetal well-being, a cervical double-balloon catheter was inserted for cervical ripening. Both balloons were inflated with 80 mL of saline according to the manufacturer’s instructions. Minimal vaginal bleeding was observed at the time of speculum removal, prompting partial deflation of the vaginal balloon. Repeat cardiotocography remained reassuring.

**Figure 1:**
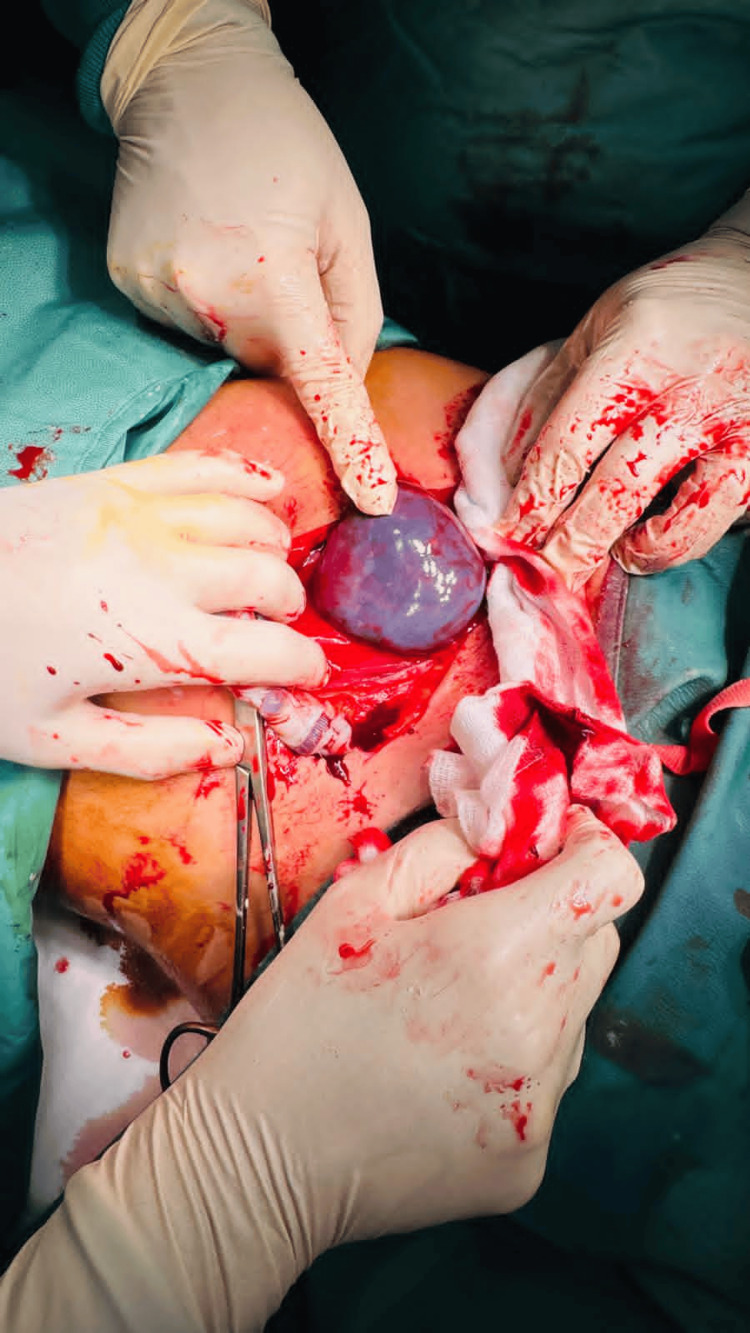
Retroplacental hematoma identified intraoperatively during the cesarean delivery.

Approximately two hours after catheter placement, the patient developed sudden-onset bright red vaginal bleeding in the absence of abdominal pain, uterine contractions, nausea, or hemodynamic instability. The balloon catheter was immediately removed, revealing significant vaginal bleeding with clots and visible placental tissue protruding through the external cervical os, consistent with partial placental prolapse. Ultrasound examination demonstrated a retroplacental hypoechoic area suggestive of an acute decidual hematoma. Abdominal palpation revealed uterine hypertonus. An emergency cesarean delivery was promptly performed. A viable neonate was delivered within eight minutes, with Apgar scores of 9 and 9 at one and five minutes, respectively. Intraoperatively, a 10-cm retroplacental hematoma was identified (Figure 1). Persistent bleeding from the placental bed was managed successfully with the placement of a Bakri balloon. Postoperative laboratory evaluation showed no indication for blood transfusion. The Bakri balloon was removed on postoperative day one. Both maternal and neonatal postoperative courses were uneventful, and the patient was discharged 72 hours after delivery.

## Discussion

Placental abruption, defined as the premature separation of a normally implanted placenta before fetal delivery, is a leading cause of vaginal bleeding in the second half of pregnancy and occurs in approximately 0.5% of pregnancies [1]. Established risk factors include advanced maternal age, smoking, cocaine use, hypertensive disorders, and a history of previous placental abruption [5,6]. Among these, preeclampsia and prior abruption confer the highest risk [1,5,6]. The association between placental abruption and preeclampsia appears strongest in cases of severe disease, whereas mild preeclampsia is only weakly associated [7]. This relationship is thought to reflect shared mechanisms of abnormal placentation early in pregnancy, leading to placental dysfunction and increased susceptibility to abruption [7]. In addition, pregnancies conceived through assisted reproductive technologies have been reported to carry an increased risk of placental abruption, estimated at approximately 83% compared with spontaneous conceptions [6]. Nonetheless, in many cases, no clear precipitating factor is identified.

The clinical presentation of placental abruption is heterogeneous. Vaginal bleeding is reported in 30-80% of cases, abdominal pain in approximately 70%, while uterine hypertonus and fetal heart rate abnormalities may be present in up to 75% of affected pregnancies [1]. Placental abruption accounts for nearly 10% of perinatal mortality [8]. Adverse neonatal outcomes are primarily related to gestational age at delivery and the timeliness of obstetric intervention, with large cohort studies demonstrating increased rates of neonatal asphyxia, respiratory distress syndrome, apnea, perinatal mortality, neonatal intensive care unit admission, and prolonged hospitalization [9]. Extensive placental separation, particularly when involving more than 50% of the placental surface, may result in fetal demise due to acute interruption of oxygen and nutrient transfer [8].

Maternal morbidity associated with placental abruption is largely driven by obstetric hemorrhage and may include hypovolemic shock requiring blood transfusion, emergency hysterectomy, disseminated intravascular coagulation, renal failure, and, in rare cases, maternal death [10,11]. Given these risks, prompt recognition and management of placental abruption are essential to optimize maternal and fetal outcomes.

In the present case, the use of a cervical double-balloon catheter may have contributed to diagnostic delay. Mechanical cervical ripening is generally considered a safe and effective method for labor induction; however, two potential concerns merit consideration. First, the mechanical forces exerted by the intrauterine balloon, including cervical dilation and separation of the fetal membranes, may theoretically precipitate placental separation in susceptible pregnancies, particularly in the presence of underlying placental pathology. Second, the presence of the balloon within the cervix and vagina may partially obscure early clinical signs of placental abruption, such as vaginal bleeding, thereby delaying recognition of the condition.

To date, only one similar case has been reported, describing placental abruption with partial placental prolapse following prolonged labor induction using a Cook balloon catheter and intravenous oxytocin in a woman with recurrent intrahepatic cholestasis of pregnancy [12]. Although causality cannot be established from isolated case reports, these observations suggest that heightened clinical vigilance may be warranted when mechanical cervical ripening is used, particularly in patients with known risk factors for placental dysfunction.

## Conclusions

Mechanical cervical ripening is widely regarded as a safe and effective method for labor induction. However, this case illustrates that the presence of a cervical double-balloon catheter may obscure early clinical signs of placental abruption, particularly vaginal bleeding, potentially delaying diagnosis. Further research is warranted to determine whether mechanical induction methods influence the timeliness of placental abruption detection and to assess whether enhanced monitoring strategies should be considered in patients with known risk factors for placental dysfunction.
